# Supplementary material for: Crystal Structure and Properties of Thallium(I) Salinomycinate
Source: Int J Mol Sci. 2025 Jul 6;26(13):6504. doi: 10.3390/ijms26136504 (PMC12249860; doi:10.3390/ijms26136504)
Supplement: Supplementary file 1 [file ijms-26-06504-s001.zip › ijms-3710315-supplementary.pdf]

International Journal of
Molecular Sciences

MDPI

Article

Thallium(I) Salinomycinate: Solid State Structure and Biological Relevance

Nikolay Petkov [1]*, Petar Dorkov [2], Angel Ugrinov [3], Elzhana Encheva [1], Miroslav Abrashev [4], Diana Zasheva [5], Teodora Daneva [5], and Ivayla N. Pantcheva [1,6]*

1 Faculty of Chemistry and Pharmacy, Sofia University "St. Kliment Ohridski", 1164 Sofia, Bulgaria; elzhanae@uni-sofia.bg
2 Research and Development Department, Biovet Ltd., 4550 Peshtera, Bulgaria; p_dorkov@biovet.com
3 Department of Chemistry and Biochemistry, North Dakota State University, Fargo ND, USA; angel.ugrinov@ndsu.edu
4 Faculty of Physics, Sofia University "St. Kliment Ohridski", 1164 Sofia, Bulgaria; mvabr@phys.uni-sofia.bg
5 Institute of Biology and Immunology of Reproduction, Bulgarian Academy of Sciences, 1113 Sofia, Bulgaria; zasheva.diana@yahoo.com (D.Z.), danevadoki@abv.bg (T.D.)
6 Centre of Competence "Sustainable Utilization of Bioresources and Waste of Medicinal and Aromatic Plants for Innovative Bioactive Products" (BIORESOURCES BG), 1164 Sofia, Bulgaria
* Correspondence: ahnp@chem.uni-sofia.bg (N.P.), ahip@chem.uni-sofia.bg (I.P.)

Supplementary information

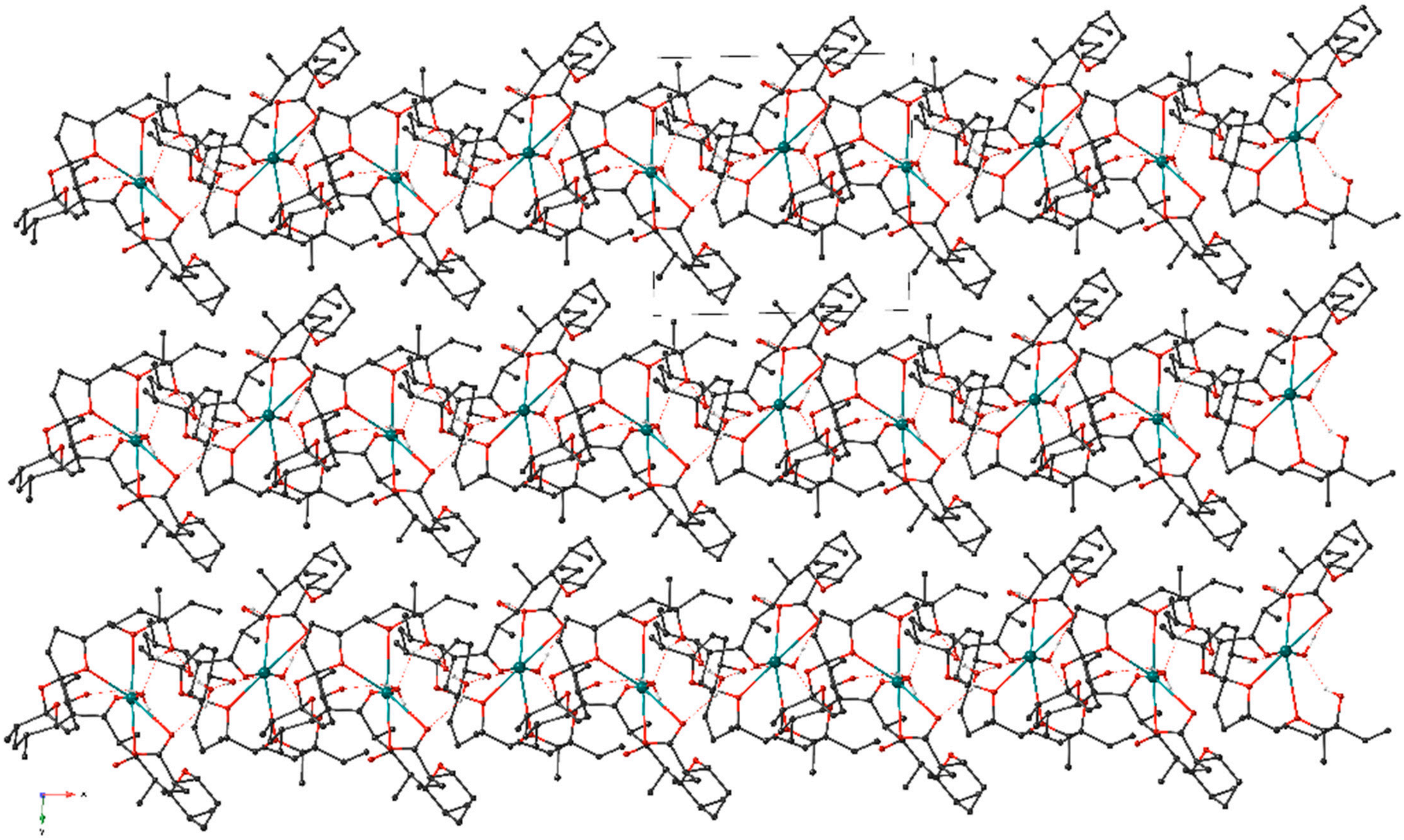

Figure S1. Crystal packing of SalTl.

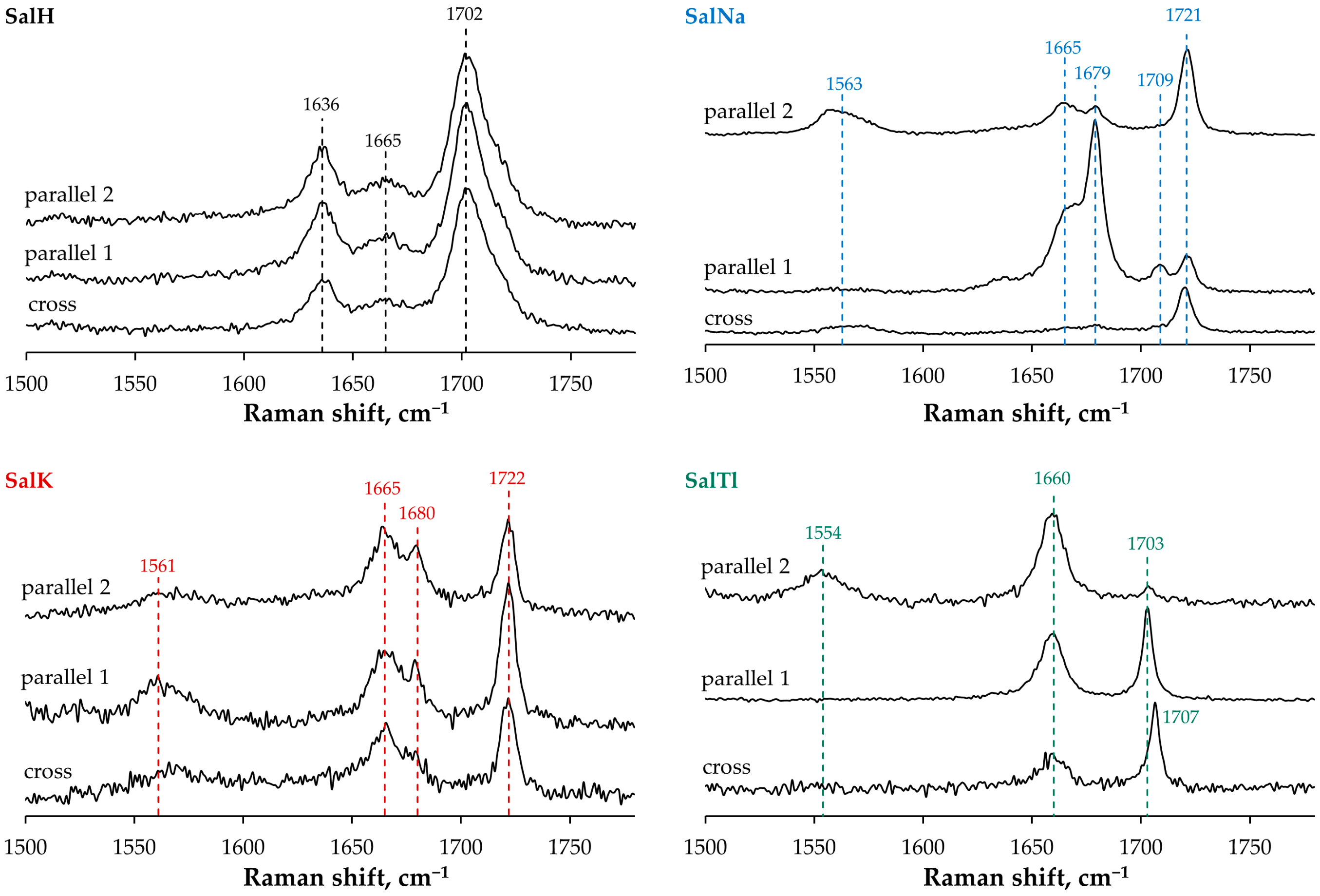

Figure S2. Comparison of the parallel and cross-polarized spectra of SalH and its complexes with Na^+, K^+ and Tl^+ ions.
